# Sodium MRI in Multiple Sclerosis is Compatible with Intracellular Sodium Accumulation and Inflammation-Induced Hyper-Cellularity of Acute Brain Lesions

**DOI:** 10.1038/srep31269

**Published:** 2016-08-10

**Authors:** Armin Biller, Isabella Pflugmann, Stephanie Badde, Ricarda Diem, Brigitte Wildemann, Armin M. Nagel, J. Jordan, Nadia Benkhedah, Jens Kleesiek

**Affiliations:** 1 Multi-Dimensional Medical Imaging Lab, Department of Neuroradiology, University of Heidelberg, 69120 Heidelberg, Germany; 2 Department of Radiology, German Cancer Research Centre (DKFZ), 69120 Heidelberg, Germany; 3 Department of Biological Psychology and Neuropsychology, University of Hamburg, 20146 Hamburg, Germany; 4 Department of Psychology, New York University, New York, NY 10003, USA; 5 Department of Neurology, University of Heidelberg, 69120 Heidelberg, Germany; 6 Molecular Neuroimmunology Group, Department of Neurology, University of Heidelberg, 69120 Heidelberg, Germany; 7 Division of Medical Physics in Radiology, German Cancer Research Centre (DKFZ), 69120 Heidelberg, Germany; 8 Department of Diagnostic and Interventional Radiology, University Medical Centre Ulm, 89070 Ulm, Germany; 9 Institute of Radiology, University Hospital Erlangen, 91054 Erlangen, Germany; 10 Multidimensional Image Processing Group, HCI/IWR, University of Heidelberg, 69120 Heidelberg, Germany

## Abstract

The cascade of inflammatory pathogenetic mechanisms in multiple sclerosis (MS) has no specific conventional MRI correlates. Clinicians therefore stipulate improved imaging specificity to define the pathological substrates of MS *in vivo* including mapping of intracellular sodium accumulation. Based upon preclinical findings and results of previous sodium MRI studies in MS patients we hypothesized that the fluid-attenuated sodium signal differs between acute and chronic lesions. We acquired brain sodium and proton MRI data of N = 29 MS patients; lesion type was defined by the presence or absence of contrast enhancement. N = 302 MS brain lesions were detected, and generalized linear mixed models were applied to predict lesion type based on sodium signals; thereby controlling for varying numbers of lesions among patients and confounding variables such as age and medication. Hierarchical model comparisons revealed that both sodium signals average tissue (χ^2^(1) = 27.89, p < 0.001) and fluid-attenuated (χ^2^(1) = 5.76, p = 0.016) improved lesion type classification. Sodium MRI signals were significantly elevated in acute compared to chronic lesions compatible with intracellular sodium accumulation in acute MS lesions. If confirmed in further studies, sodium MRI could serve as biomarker for diagnostic assessment of MS, and as readout parameter in clinical trials promoting attenuation of chronic inflammation.

## Introduction

Multiple sclerosis (MS) is an inflammation-mediated disease of the CNS^1^. The genesis of MS is characterized by a cascade of pathologic events, including focal lymphocytic infiltration, microglia activation, demyelination, and axonal degeneration^2^. A potentially important inflammatory mechanism leading to axonal degeneration is the production of reactive-oxygen species and nitric oxide from activated microglia and infiltrated macrophages^3^,^4^. Reactive-oxygen species and nitric oxide promote mitochondrial injury^5^,^6^. Impaired neuronal mitochondrial function, in turn, induces additional oxidative stress by increased production of reactive-oxygen species^7^, and results in reduced ATP production^8^,^9^. Concurrently, there is an increased energy demand due to the re-organization of sodium channels, which occurs in response to axonal demyelination^10^,^11^. The neuronal energy deficit leads to an intracellular accumulation of sodium ions^12^, and thereby promotes the sodium-calcium exchanger to operate in reverse^13^. The resulting intracellular culmination of calcium can contribute to further mitochondrial damage and activation of nitric oxide synthase, proteases as well as lipases^3^ – a cascade eventually resulting in neuronal cell death^9^.

The cascade of pathogenetic mechanisms in MS has no specific conventional MRI correlates. Imaging specificity needs to be improved to define the pathological substrates of MS *in vivo*^14^. Based on the reported effects of MS on sodium channels, sodium MRI seems promising for this endeavour: Inglese and colleagues^15^ and Paling and colleagues^16^ showed that the average tissue sodium signal is elevated in MS lesions compared to normal-appearing white matter, and increased in normal-appearing white matter of MS patients compared to healthy controls. Also, the two groups revealed a correlation between sodium signal strength and disability status. However, both intra- and extracellular sodium ions contribute to this overall signal and therefore changes cannot be attributed to a certain microstructural compartment. Consequently, one long-term goal in understanding the mechanisms of MS is to distinguish intracellular from extracellular sodium ions^3^. In this study, we propose to include an additional, so-called fluid-attenuated sodium signal (also known as intracellular-weighted or relaxation-weighted sodium signal), which allows further information on the origin of signal alterations. As this technique emphasizes sodium ions with short relaxation times a weighting towards the intracellular sodium compartment is achieved^17^,^18^,^19^. Similar to our approach, Madelin and colleagues recently applied a sodium MR sequence to estimate the intracellular sodium concentration of healthy brain tissue^20^; sequence details are discussed elsewhere^21^. Further, it has been shown in brain tumours, that the fluid-attenuated sodium signal adds significant information to the average tissue sodium signal based on a strong correlation between fluid-attenuated sodium signal and the proliferation rate of tumour cells^21^,^22^.

On the basis of preclinical work^1^,^4^,^5^,^8^,^9^,^12^,^13^ and sodium MRI findings in MS^15^,^16^,^23^,^24^, we postulated that the fluid-attenuated sodium signal should differ between acute and chronic lesions, and, thus, provide additional information on lesion type in MS. Therefore, we measured both average tissue and fluid-attenuated sodium signals of brain tissue lesions in MS, and compared these measures between acute and chronic lesions as defined by the actual reference standard, which is the presence or absence of contrast-enhancement in canonical T1-weighted images. Additionally, we were able to record signals from three patients with acute lesions before and after drug based treatment.

## Methods

### Ethics statement

The study was approved by the local Medical Ethics Committee (Faculty of Clinical Medicine, University of Heidelberg). All participants provided written informed consent prior to enrolment. The procedures that follow were in accordance with the declaration of Helsinki.

### Participants and study design

In this study, we enrolled patients with the diagnosis of MS, and acute and/or chronic brain lesions. Eligible patients had no history of head trauma, no vascular pathologies or medical conditions possibly leading to brain lesion formation other than MS, and no contraindications to ultra-high field MRI. Diagnosis of MS was made according to the 2005 McDonald criteria^25^. The course of disease was defined according to Lublin and Reingold^26^: A secondary-progressive MS was diagnosed if a deterioration of clinical disability independent of relapses exists for at least six months following an initial relapsing-remitting MS course. Detailed epidemiologic patient data are shown in Table 1.

### Procedures

#### MR imaging

##### Cross-sectional experiment

For lesion detection, clinical routine proton MRI data were acquired using a 3 Tesla whole-body system (Tim Trio 3T, Siemens Healthcare, Erlangen, Germany) with T2-FLAIR, T2-TSE, native and contrast-enhanced T1-3D ultrafast gradient sequences. Sodium MRI was performed using a 7 Tesla whole-body MR system (Magnetom 7T, Siemens Healthcare, Erlangen, Germany) and a double-resonant (^1^H/^23^Na) quadrature birdcage coil with an inner coil diameter of 26 cm (Rapid Biomed GmbH, Rimpar, Germany). All sodium MR sequences were based on a 3D density-adapted projection reconstruction technique^27^ providing an average tissue sodium signal, and a fluid-attenuated sodium signal. Weighting in this context means that extracellular sodium may contribute to the fluid-attenuated signal. There is no quantitative information of how strong the weighting is. Sequence details are shown in Table 2.

##### Longitudinal experiment

Three patients (PIDs no. 15, 21 and 29; cf. Table 1) of the cross-sectional experiment were additionally measured after administration of high-dose methylprednisolone using the identical MR imaging protocol. Thus, in these patients acute MS lesions could be longitudinally monitored before and after steroid therapy.

#### Image processing

**Figure 1:**
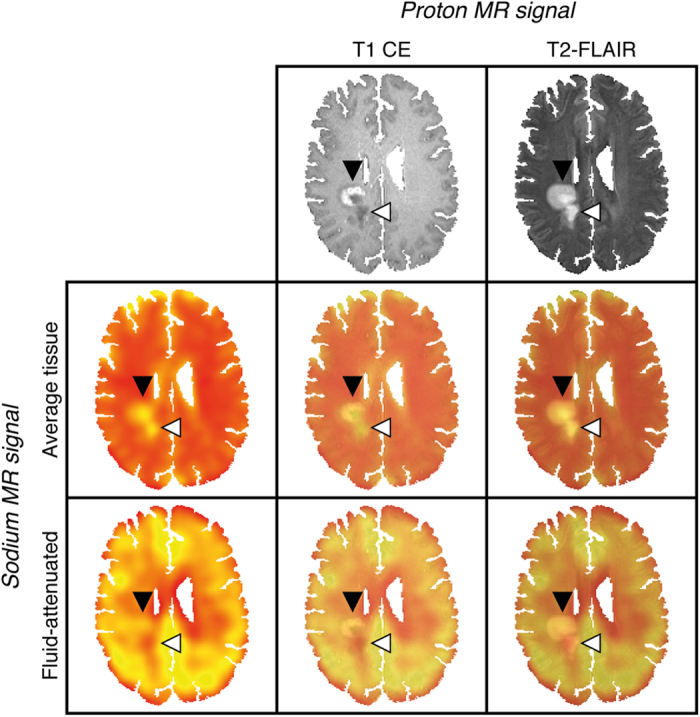
Exemplary co-registered sodium and proton images of a patient with acute MS lesions (PID no. 15). Proton MRI demonstrates two (confluent) right-central white matter lesions. The anterior lesion exhibits contrast-enhancement and corresponding elevated average tissue and fluid-attenuated sodium signals compatible with acute inflammation (black arrowhead). The posterior lesion shows no signs of blood-brain barrier disruption, an increased average tissue sodium signal and a reduced fluid-attenuated sodium signal – a combination consistent with the residuals of brain tissue inflammation (white arrowhead). This representative example demonstrates high intermodal registration accuracy of the applied affine image transformation method (FLIRT); T1 CE, T2-FLAIR and sodium images as well as the corresponding overlays are shown.

Sodium image reconstruction was performed offline by a custom-written MATLAB script (The Mathworks Inc, Natick, MA, USA). To reduce Gibbs ringing artifacts, a Hamming filter was applied. Contrast-enhanced T1-weighted images (T1 CE) were skull-stripped by the Brain Extraction Tool (BET, part of FMRIB’s Software Library FSL)^28^ and served as individual reference images. T2-TSE, T2-FLAIR, and sodium images were co-registered to this reference image using an affine registration with 12 degrees of freedom as implemented in FMRIB’s Linear Image Registration Tool (FLIRT, part of FSL)^29^. Figure 1 shows an example of co-registered sodium and proton MR images. Sodium data were normalized to healthy parenchyma, i.e., to brain tissue without T2 signal alterations and without pathological contrast enhancement in the T1 signal: For every individual and each MR sequence, we performed a voxelwise division of signal values from identified lesions by the respective mean signal of healthy tissue. Normalizing the data aimed at the reduction of inter-individual variance in the sodium MR signal, and at the comparability of imaging data among subjects. All values are in arbitrary units.

#### Multiple sclerosis lesions

MS lesion volumes of interest were defined by T2-FLAIR signal alterations and, where applicable, by the T1 signal of contrast-enhancing portions. Thereby, the presence (acute lesion) or absence of contrast enhancement (chronic lesion) determined the type of lesion. Lesions less than 30 mm^2^ in volume, and less than 5 mm in transversal diameter were discarded from further analysis to reduce partial volume effects. All included lesions were located within white matter. Outlier corrections removed data diverging by 2.5 standard deviations from the intra-individual mean value of all lesions. Plausibility checks controlled for T1 CE signals to be higher in acute compared to chronic lesions; lesions not fulfilling these criteria were removed from further analysis.

### Statistical analysis

#### Prediction of lesion type

For each lesion its type (acute vs. chronic) was predicted using generalized linear mixed models with random intercepts, which allow controlling for varying numbers of lesions and lesion types between participants. The predictive value of average tissue sodium signal and fluid-attenuated sodium signal was explored by adding these predictors successively to a base model and evaluating whether these led to an improvement of the model which outweighed the additional number of free parameters using likelihood ratio tests. Several other factors possibly associated with lesion type were controlled for by adding them as predictors to all models. In detail, age, gender, methylprednisolone application, medication other than methylprednisolone, expanded disability status scale (EDSS) score, and disease duration were included in all models. Model parameters were estimated using Laplace approximation as implemented in the lme4 package^30^ in R. To ease convergence of the algorithm all continuous predictors were scaled, i.e., z-transformed prior to parameter estimation.

#### Correlation analyses

As for the prediction of lesion type a hierarchical design was used to test for the relationship between sodium and proton MRI signals, thereby, controlling for differences in the number of lesion (types) across participants. To ensure continuity with the previous analysis we calculated linear mixed models, predicting separately native T1, T1 CE, T2-TSE, and T2-FLAIR from average tissue and fluid-attenuated sodium signals. All signals were standardized beforehand, thus, the estimated regression parameters correspond to Pearson’s correlation coefficient. Note that due to the hierarchical design fixed effects parameters are free of inter-subject variability in the strength of the signal, thus, being higher than suggested from a scatterplot across all data points. Therefore, scatterplots are not shown here. Statistical significance of the estimated parameter values was tested using type III Wald chi-square tests.

#### Longitudinal data

This analysis aimed at revealing changes in sodium MRI signals after steroid therapy. To this aim, average tissue and fluid-attenuated sodium signals were separately predicted by the timepoint of signal measurement. This analysis compares signal values between pre- and post-medication states while accounting for differences in lesion number and average signal strength in single patients. A statistical model based on data from three patients cannot be representative with regard to the whole patient group. To derive a measure of confidence for parameter estimates from this model we performed 100,000 parametric bootstraps of each model.

## Results

### Cross-sectional experiment

#### Lesion segmentation

N = 330 supratentorial parenchymal lesions were detected and thereby classified as acute (n = 82) or chronic (n = 248) depending on the presence or absence of pathologic contrast enhancement. Outlier corrections and plausibility checks (cf. Methods) reduced the number of lesions leaving n = 302 lesions including n = 70 acute and n = 232 chronic lesions for further analysis.

#### Prediction of lesion type

**Figure 2:**
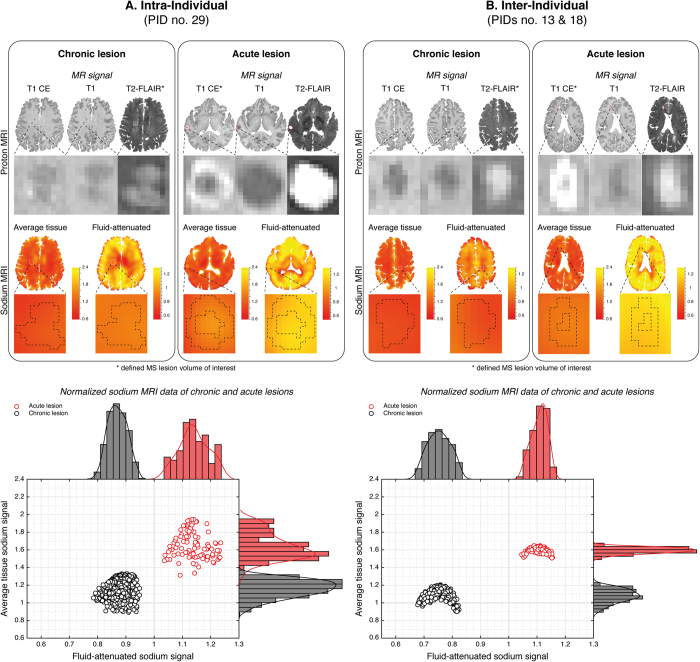
Exemplary intra- and inter-individual sodium MRI data of chronic and acute MS lesions. Intra-individual (A; PID no. 29) proton MRI shows a T2-hyperintense chronic lesion of the right-frontal parenchyma (**A**), left panel; lesion no. 2) without contrast-enhancement (CE) and an acute T2-hyperintense, right-temporal lesion with associated CE (A, right panel; lesion no. 2). Similar, inter-individual (B; PIDs no. 13 and no. 18) proton MRI shows a T2-hyperintense chronic lesion of the left-central parenchyma (**B**), left panel; lesion no. 2) without contrast-enhancement (CE) and an acute T2-hyperintense, right fronto-mesial lesion with associated CE (B, right panel; lesion no. 5). In acute lesions the (intra- and inter-individual) average tissue and the fluid-attenuated sodium signals are increased compared to chronic lesions. Scatter plots and histograms of both sodium signals visualize intra- and inter-individual examples with ideal separation of acute and chronic lesions by average tissue and fluid-attenuated sodium signals; this is not true for all lesions, that is, scatter plots of MS lesions can be larger, and may be somewhat overlapping between lesion types. This may be, at least in part, owed to the fact that both, T2 signal and contrast enhancement are primarily unspecific^14^,^43^. Nevertheless, generalized linear mixed models revealed a significant improvement of lesion type classification by the average as well as the fluid-attenuated sodium signal. Both signals were significantly increased in acute lesions. (All values are in arbitrary units; dashed lines projected onto the zoomed sodium images indicate the morphological outlines as defined by proton MRI; please note however, that these outlines are not necessarily identical to sodium MRI signal alterations of these lesions; thick gray grid lines represent the average tissue (y-axis) and fluid-attenuated sodium signal values (x-axis) of healthy parenchyma)

MS lesions were analysed by generalized linear mixed models, which revealed that both, average tissue and fluid-attenuated sodium signals significantly improved lesion type classification (average tissue sodium signal: χ^2^(1) = 27.89, p < 0.001; fluid-attenuated sodium signal: χ^2^(1) = 5.76, p = 0.016). The average, as well as the fluid-attenuated sodium signal of acute MS lesions were thus significantly elevated compared to chronic MS lesions. In Fig. 2, these results are exemplarily visualized for intra- and inter-individual MS lesions.

#### Correlation analyses of sodium and proton MR signals

Hierarchical analyses of sodium and proton MRI signals in MS lesion volumes of interest revealed a weak correlation of the average tissue sodium signal with the T2-FLAIR signal (β = 0.38 (0.09), χ^2^ = 18.86, p < 0.001), but indicated strong correlations of the average tissue sodium signal with T2-TSE (β = 0.90 (0.07), χ^2^ = 147.04, p < 0.001) and native T1 signals (β = 0.92 (0.10), χ^2^ = 86.75, p < 0.001). There was no correlation between average tissue sodium signal and T1 CE signal.

In addition, statistical analyses detected a correlation of fluid-attenuated sodium signal and T1 CE signal (β = 0.34 (0.08), χ^2^ = 17.94, p < 0.001). There were no associations of the fluid-attenuated sodium signal with T2-FLAIR, T2-TSE, and native T1 signal. Of note, there was no correlation between average tissue and fluid-attenuated sodium signals.

### Longitudinal experiment

**Figure 3:**
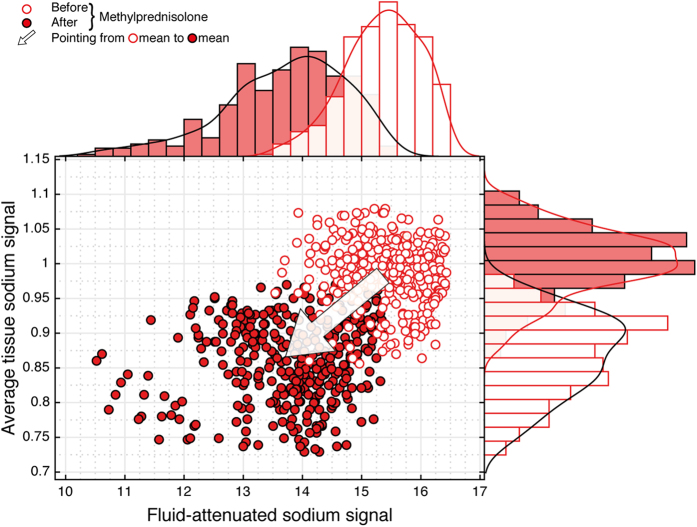
Longitudinal sodium MRI data of an acute lesion (PID no. 15). After application of high-dose methylprednisolone both the average tissue and fluid-attenuated sodium signal decrease. Based upon the cross sectional findings on sodium signal differences between acute and chronic MS lesions, this signal behavior was proposed before. Moreover, it strongly supports the notion that findings of our study are compatible with intracellular sodium accumulation in acute inflammatory MS lesions. Please note, that due to the pharmacological intervention changes in tissue sodium concentration of healthy parenchyma could not be ruled out. Thus, for the longitudinal observations, normalization referred to transmitter amplitudes instead of the sodium signal of healthy parenchyma.

**Figure 4:**
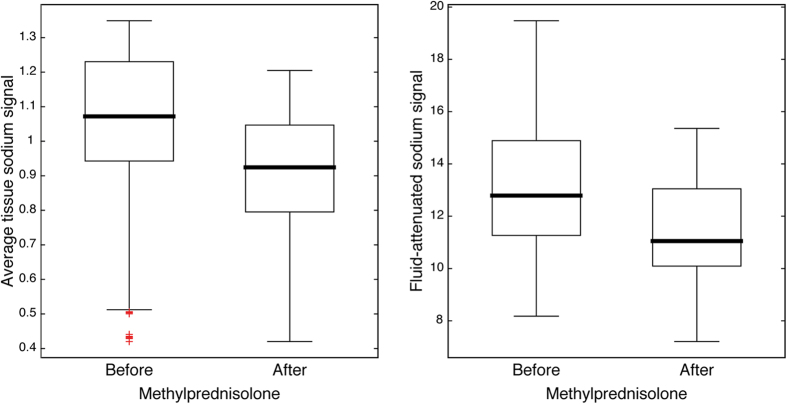
Longitudinal monitoring of all acute MS lesions (PIDs no. 15, 21 and 29). Sodium signal changes after administration of high-dose methylprednisolone support findings of the cross-sectional analyses. Hierarchical comparisons of average tissue and fluid-attenuated sodium signals across pre- and post-medication measurements revealed a decrease of both signals after the application of high-dose methylprednisolone (cf. Methods and Results section for details).

Hierarchical comparisons of average tissue and fluid-attenuated sodium signals across pre- and post-medication measurements revealed a decrease of both signals after the application of high-dose methylprednisolone. Average tissue sodium signals decreased by estimated 0.07 a.u. ([−0.18, 0.03]; 95% confidence interval based on parametric bootstrapping). Fluid-attenuated sodium signals even decreased by 1.45 a.u. [−2.51, −0.38] after medication. Longitudinal sodium MRI data are shown in Figs 3 and 4.

## Discussion

Our study confirms that both, the average tissue sodium signal and the fluid-attenuated sodium signal significantly differ between acute and chronic MS lesions of human brain parenchyma. Additional longitudinal sodium data from before and after application of high-dose methyprednisolone demonstrate that these differences are MS specific. Therefore, the detected increase in fluid-attenuated sodium signal is compatible with the intracellular sodium accumulation observed in acute MS lesions. This finding is in excellent agreement with previous reports on increased expression of sodium channels, and intracellular sodium accumulation occurring as pathophysiological event in experimental autoimmune encephalomyelitis (EAE) and MS^11^,^12^,^31^. Moreover, consistent with results of our study, Petracca and colleagues^32^ demonstrated sodium signals at a brain regional level that might reflect neuro-axonal metabolic dysfunction in MS.

**Figure 5:**
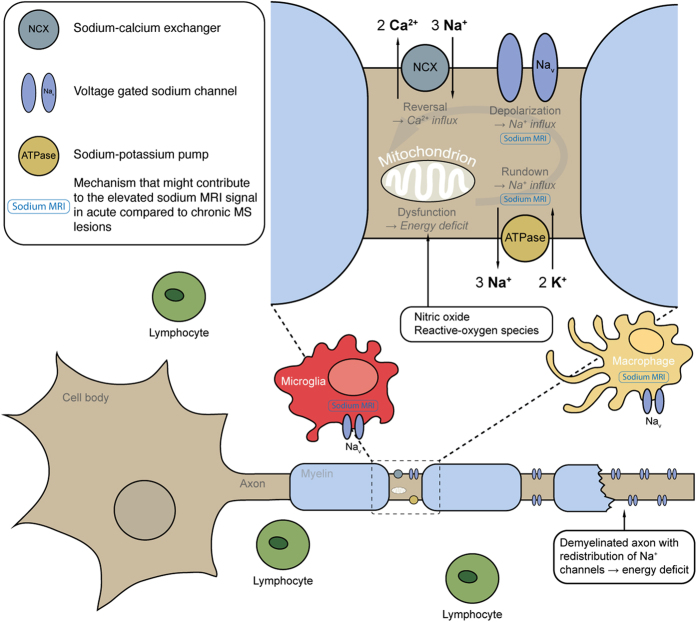
Overview of the pathological mechanisms in MS that might contribute to sodium MRI signal elevation in acute lesions. The pathogenesis of MS involves a cascade of pathological events, including focal lymphocytic infiltration, microglia and macrophage activation, demyelination, and axonal degeneration^2^. Na_v_1.6 is the predominant sodium channel expressed in microglia and macrophages, and is up regulated upon activation of these cells in EAE and acute MS lesions^35^,^36^. Activated microglia and macrophages produce reactive-oxygen species^4^ and nitric oxide^3^ and thus induce mitochondrial dysfunction in neurons^37^,^38^ with subsequently reduced energy supply. Concurrently, re-distribution of Na_v_1.6 along demyelinated axons in EAE^31^ and acute MS lesions^11^ increases energy demand. The mismatch between reduced energy supply and elevated demand creates a virtual hypoxia^39^, which also impairs the sodium-potassium pump^9^, and leads to an intracellular accumulation of sodium ions^12^. This ionic imbalance promotes the sodium-calcium exchanger to operate in reverse^13^. Consecutive calcium import leads to injurious intracellular calcium levels with deleterious sequelae to the neuron^9^. Mechanisms of MS associated with intracellular sodium accumulation that possibly contribute to elevated average and fluid-attenuated sodium MRI signals are marked in blue. Also, hyper-cellularity itself might cause an increased fluid-attenuated signal. In fact, it may well be that both, intracellular sodium accumulation and hyper-cellularity contribute to the increased sodium signals observed in acute MS lesions as both are part of the same pathophysiologic mechanism.

In Fig. 5, our results and recent preclinical findings are put into context. There is strong evidence that sodium channels play an important role in immune cell function in EAE and MS^11^,^31^. Na_v_1.6 is the predominant sodium channel expressed in microglia and macrophages^33^,^34^. Its expression is up regulated on activation of these cells in EAE and acute MS lesions^35^,^36^. In MS lesions, activated microglia and infiltrated macrophages^4^ produce reactive-oxygen species and nitric oxide^3^, which impair mitochondrial function in neurons^37^,^38^. Thereby, ATP production and energy supply is reduced. Concurrently, there is an increased neuronal energy demand due to the redistribution, as well as the co-localization of Na_v_1.6 and the sodium-calcium exchanger along demyelinated axons in EAE^31^ and in acute MS lesions^11^. This mismatch between energy supply and demand thereby creates a state of virtual hypoxia^39^, and leads to an accumulation of intracellular sodium ions^12^ similar to the pathogenic cascade following ischemic stroke^40^. The ionic balance changes promote the sodium-calcium pump to operate in reverse^13^, i.e. to import calcium into the cell. This leads to injurious calcium levels, and eventually to the activation of proteases, lipases, and nitric oxide-synthase as well as to mitochondrial damage, and ultimately to neuronal cell death^9^. The role of sodium channels in immune cell function is further emphasized by the fact that blocking sodium channels with phenytoin reduces inflammatory cell infiltration in MS lesions^35^. Also, with safinamide and flecainide, two other sodium channel blockers, activation of microglia and macrophages is suppressed and symptoms are ameliorated in EAE^36^.

Theoretically, longitudinal relaxation times of sodium ions within the cell resemble those in hyper-cellular environment. Therefore, increased fluid-attenuated sodium signals of acute MS lesions might as well originate, at least in part, from hyper-cellularity related to inflammation; that is to accumulating immune cells like activated microglia or infiltrated macrophages. In fact, it may well be that both, intracellular sodium accumulation and hyper-cellularity contribute to the increased sodium signals observed in acute MS lesions as both are part of the same pathophysiologic mechanism.

Inglese and colleagues^15^ and Paling and colleagues^16^ showed that the average tissue sodium signal is elevated in MS lesions compared to normal-appearing white matter, and increased in normal-appearing white matter of MS patients compared to healthy controls. In addition to the average tissue sodium signal, in the study presented here we acquired a fluid-attenuated sodium signal. Correlation analyses revealed an association between fluid-attenuated sodium and T1 CE signals, but not between average tissue sodium and T1 CE signals; the average tissue sodium signal strongly correlated with the T2-TSE and native T1 signals whereas the fluid-attenuated sodium signal did not. In addition, there was no correlation between average tissue and fluid-attenuated sodium signals – facts that underline the additional information of the fluid-attenuated sodium signal. The fluid-attenuated signal provides new biological information on MS lesions. Thereby, it allows for a more detailed interpretation of the average tissue sodium signal increase previously reported in MS lesions, which might be owed to increased extracellular space by neuroaxonal loss^41^ or vasogenic oedema^42^, or to intra-axonal sodium accumulation^12^.

In a longitudinal experiment, we monitored the sodium signals before and after administration of high-dose methylprednisolone and found a significant decrease in average tissue and fluid-attenuated sodium signal of acute lesions following anti-inflammatory pharmacologic intervention (Figs 3 and 4). Thereby, the longitudinal findings support the cross-sectional results of this study. They indicate that sodium signal differences between acute and chronic MS lesions are indeed attributable to inflammatory mechanisms. Moreover, results are in agreement with the cascade of those pathobiological events in MS that are associated with intracellular sodium increase, and eventually lead to lesion formation.

The spatial resolution of sodium MRI is limited, and therefore partial volume effects may have lowered sensitivity in detecting signal changes especially in very small inflammatory lesions. Still, statistical analyses indicate sodium signal changes large enough to predict lesion type. In future, technical advances like iterative image reconstruction techniques and multi-channel array coils will improve the spatial resolution, and, thereby facilitate the analysis of small lesions.

In this study, MS lesions were identified by their T2 signal; they were then defined as acute or chronic depending on the presence and absence of contrast enhancement in T1-weighted images, respectively. Although this approach is gold standard in diagnostic imaging of MS to date it most probably introduces uncertainty into sodium MRI based lesion type prediction because lesion definition relies on T2 signal as well as contrast enhancement, and both are principally unspecific^14^,^43^; that is, for example, T2-hyperintense lesions may represent acute inflammation although no contrast enhancement is detectable^44^. Despite this possible unexplained variance in sodium MRI data, again, significant sodium signal differences between acute and chronic MS lesions could be detected in the study presented here. Invasive studies would be necessary to confirm whether sodium imaging is able to classify lesions not classified by proton MRI.

To our knowledge, this is the first *in vivo* study that discloses significant sodium signal differences between acute and chronic MS lesions of the brain. The fluid-attenuated sodium signal reveals new biological information on lesion evolution, which is attributable to inflammatory mechanisms and compatible with those pathogenetic events of MS leading to intracellular sodium accumulation, hyper-cellularity and neurodegeneration. If confirmed in other studies, the average tissue and the fluid-attenuated sodium signal could be considered as neuroimaging biomarkers for diagnostic assessment and readout parameter for therapy monitoring, and complement clinical outcome measures. Together with the excellent spatial tissue characterization of proton MRI, the biological tissue information by sodium MRI might promote the endeavors to enhance imaging specificity for defining the pathological substrates of MS *in vivo* – a long-term goal as suggested by experts in the field of neurology^3^,^14^,^45^. In addition, the combination of proton (lesion identification) and sodium (lesion differentiation) MRI appears to be a potential alternative to contrast media administration in the assessment of MS; avoiding gadolinium-based contrast agents is advocated with respect to unwanted gadolinium accumulation in brain tissue^46^,^47^,^48^,^49^. This is especially relevant for MS patients as they rely on continuous follow-up MRI including gadolinium administration.

## Additional Information

**How to cite this article**: Biller, A. *et al*. Sodium MRI in Multiple Sclerosis is Compatible with Intracellular Sodium Accumulation and Inflammation-Induced Hyper-Cellularity of Acute Brain Lesions.. *Sci. Rep.* **6**, 31269; doi: 10.1038/srep31269 (2016).

## Figures and Tables

**Table 1:** Patient characteristics.

Patient ID	Age (years)	Gender	Stage	EDSS	MS lesion type		Methyl-prednisolone		Medication other than methyl- prednisolone	Disease duration (months)
					*Acute*	*Chronic*	*Prior to MRI*	*At the time of MRI*	*At the time of MRI*	
1	27	Female	RRMS	2		X			Interferon beta	15.05
2	42	Male	RRMS	2		X		X	None	0.30
3	20	Male	RRMS	0	X*				Fingolimod	10.02
4	18	Male	RRMS	2		X			Interferon beta	9.63
5	58	Male	RRMS	3		X			Glatiramer ac.	412.55
6	33	Male	RRMS	1.5	X	X			Glatiramer ac.	44.05
7	25	Male	RRMS	0	X*		X		None	5.55
8	50	Male	RRMS	0		X			Natalizumab	96.62
9	50	Female	RRMS	4	X*	X	X		None	66.06
10	49	Male	RRMS	1.5		X			Dimethyl fum.	231.67
11	47	Female	RRMS	4		X			Interferon beta	212.32
12	39	Female	RRMS	2	X		X		None	0.03
13	34	Female	RRMS	NI	X	X	X		NI	NI
14	52	Female	RRMS	3	X	X			Glatiramer ac.	101.08
15	48	Female	RRMS	2	X	X	X		None	0.03
16	50	Female	RRMS	5	X		X		None	0.53
17	50	Female	SPMS	5.5		X			Glatiramer ac.	103.84
18	25	Female	RRMS	1		X			Fingolimod	102.83
19	23	Female	RRMS	0		X			Fingolimod	30.09
20	40	Male	RRMS	3		X			Interferon beta	28.91
21	25	Female	RRMS	2	X	X	X		Interferon beta	34.76
22	43	Male	RRMS	2	X	X			Interferon beta	24.80
23	27	Female	RRMS	2	X*	X	X		None	4.07
24	45	Female	RRMS	4	X	X			Fingolimod	210.91
25	34	Female	RRMS	1	X	X			Interferon beta	43.36
26	37	Male	RRMS	2.5		X			Interferon beta	73.23
27	36	Male	RRMS	1	X	X			None	0.99
28	60	Male	RRMS	1.5		X			None	49.87
29	34	Male	RRMS	2	X	X	X		None	110.32

RRMS = relapsing-remitting multiple sclerosis; SPMS = secondary progressive multiple sclerosis; *acute lesion of the spinal cord, no brain lesion; ac. = acetate; fum. = fumarate; NI = no information available.

**Table 2:** Sodium MRI sequence details.

	Sodium MR Sequence	
	Average tissue sodium MRI signal^*^	Fluid-attenuated sodium MRI signal**
**Echo Time (TE)**	0.35 ms	0.75 ms
**Repetition Time (TR)**	160 ms	185 ms
**Readout Duration (T**_**RO**_)	10 ms	16.7 ms
**Inversion Time (TI)**	—	41 ms
**Nominal Spatial Resolution**	3.0 × 3.0 × 3.0 mm^3^	4.4 × 4.4 × 4.4 mm^3^
**Acquisition Time**	10 min 4 s	9 min 52 s

*We minimized relaxation weighting by a short TE and a long TR; **We measured the fluid-attenuated sodium signal using an inversion recovery sequence which suppresses signal of sodium ions with long relaxation times as, for example, in cerebro-spinal fluid.
